# Cognition is only minimally impaired in Spinocerebellar ataxia type 14 (SCA14): a neuropsychological study of ten Norwegian subjects compared to intrafamilial controls and population norm

**DOI:** 10.1186/1471-2377-13-186

**Published:** 2013-11-29

**Authors:** Iselin Marie Wedding, Jeanette Koht, Espen Dietrichs, Nils Inge Landrø, Chantal ME Tallaksen

**Affiliations:** 1 Department of Neurology, Oslo University Hospital, Ullevål, Oslo, Norway; 2 Faculty of Medicine, University of Oslo, Oslo, Norway; 3 Clinical Neuroscience Research Group, Department of Psychology, University of Oslo, Oslo, Norway; 4 Department of Neurology, Drammen Hospital, Vestre Viken Health Trust, Drammen, Norway

**Keywords:** Ataxia, SCA14, Cerebellum, Cognition, Protein kinase C γ

## Abstract

**Background:**

There is an increasing awareness of the role of the cerebellum not only in motor, but also in cognitive and emotional functions. Spinocerebellar ataxia type 14 (SCA14) is an autosomal dominant hereditary ataxia characterized by a relatively pure cerebellar phenotype. Cognitive impairment has been reported in studies with phenotype descriptions of SCA14, but previous studies have been small without control groups, and no homogeneous and systematic test panel has been used. The objective of this study was to thoroughly characterize the neuropsychological profile in ten Norwegian SCA14 subjects compared to unaffected family members and population norm data.

**Methods:**

Ten SCA14 subjects and ten intrafamilial unaffected age- and education-matched controls from two Norwegian families were included. The unaffected intrafamilial controls included six first degree relatives, two second degree relatives, and two spouses. General intellectual ability, memory, visuoperceptive skills, psychomotor speed, executive functions, depression and anxiety were examined using internationally standardized tests, with minimal need for manual response to avoid motor bias.

**Results:**

No significant cognitive deficit was found in SCA14 subjects compared to intrafamilial controls. Verbal IQ, verbal executive function and psychomotor speed tended to be reduced in affected subjects, but previously reported non-verbal executive dysfunction was not confirmed in this study.

**Conclusion:**

Only subtle cognitive impairment was found in SCA14 affected subjects. The current findings do not confirm earlier reports of cognitive dysfunction in SCA14, but does shows a mild impairment in specific verbal executive functions. Genotypic differences may partly account for this discrepancy, and further studies on larger materials are needed to verify the findings.

## Background

Cerebellum is traditionally viewed as being mainly an important part of the motor system in the coordination of movement and motor learning. During the last two decades there has been a growing interest for the cerebellum’s role also in non-motor functions, with the introduction of the “dysmetria of thought”-hypothesis and the clinically described Cerebellar Cognitive Affective Syndrome (CCAS) [1], consisting of executive dysfunction, visuospatial and verbal impairment and affective dysregulation. In addition, neuroanatomical [2], evolutionary [3-5] and functional imaging [6,7] findings support a role of the cerebellum in cognition.

Autosomal dominant cerebellar ataxias (ADCAs) are neurodegenerative inherited disorders with progressive ataxia. Additional signs and symptoms may be present, such as polyneuropathy, pyramidal signs or cognitive impairment. Genotypically, ADCAs may present with conventional mutations or expansions. There appears to be a marked difference in the way the two categories of mutations affect the extracerebellar domains [8]. Conventional mutation ADCAs tend to present with a pure cerebellar phenotype with marked isolated cerebellar atrophy. Most studies of cognition in ADCAs have been done in polyglutamine expansion SCAs and show a wide variety of cognitive deficits [9-15]. Fewer studies have been done in ADCAs with conventional mutations, but the cognitive profile in these ADCAs should be particularly interesting, as impairment is expected to result from a relatively pure cerebellar dysfunction.

Spinocerebellar ataxia type 14 (SCA14, OMIM; Mendelian Inheritance in Man 605361) is characterized by a mild and slow neurodegeneration with onset between 10–60 years, and with cerebellar symptoms and clinical cerebellar signs. The disorder is described as rare, representing 1.5-4% of the European ADCA families [16,17]. SCA14 shows a relatively pure cerebellar phenotype [18-20], though minor extracerebellar symptoms and signs may be present. The disease is associated with point mutations in the *PRKCG* gene which is expressed throughout the central nervous system, most prominently in Purkinje cells. According to the theory of cerebellar somatotopy [21], cognitive and affective functions have been related to the posterior lobe and vermis especially in the lateral regions of lobule VI and VII (Crus I, Crus II, and VIIB) [6]. Radiologically, cerebellar atrophy is reported in SCA14 patients and is most pronounced in the vermis and posterior lobe [19,22-24], and one could thus expect vulnerability for non-motor symptoms in SCA14 patients.

Cognitive impairment has been reported in studies with phenotype descriptions of SCA14 (Table 1) [17,25-27], but detailed cognitive evaluation is only reported in a few cases, with no control groups [18]. Our primary objective was to systematically investigate cognitive impairment in SCA14 as compared to intrafamilial controls and norm data. As a secondary aim we wanted to assess the impact of motor symptoms severity and disease duration on neuropsychological performances.

**Table 1:** Earlier reports of cognitive function in SCA14

**Author**	**IQ**	**Number of patients**	**Other cognitive features**
Klebe et al. [17]	1 tested - normal global IQ	15	*Patient 1:* deficits in abstract thinking and shifting, verbal fluency and inhibitory control, subcortical dementia.
		2 patients extensively tested	*Patient 2:* memory deficit and impaired executive functions, impaired working memory, increased sensibility to proactive interference in memory, tendency to perseverate. 2 out of 15 characterized as demented on clinical impression only
Stevanin et al. [27]	4 out of 7 indications of low IQ and difficulty with abstract thinking*	18	*Patient 1:* executive function deficit, difficulties with memory encoding and retrieval, attention deficit, cognitive slowing, impaired working memory, concept shifting, abstract thinking, resistance to interference and inhibitory control
		2 patients extensively tested	
			*Patient 2:* impaired executive functions, tendency to perseverate, attention deficit, lack of inhibitory control
Hiramoto et al. [25]	n.a.	5	1 patient described with mental retardation, 4 patients described with no intellectual disturbance
Miura et al. [26]	Total IQ (66,73,93), verbal IQ (79,77,90), performance IQ (58,74,99)	3	Visual memory deficit in 1

*Evaluated by Progressive Matrix 47.

## Methods

### Participants

#### Subjects

Two SCA14 families were identified during an ongoing project on hereditary ataxias in Norway [19,28]. Ten mutation carriers, nine from family 1 and one from family 2 (Table 2), were initially included in the study as affected subjects, independently of clinical signs and symptoms. This group included seven males and three females, with an age range between 17 and 54 years. They represented all identified subjects with SCA14 above the age of 16 in Norway at the time of the study, with the exception of one subject who had suffered a stroke and could therefore not participate. One subject (Subject V10) was confirmed to have the mutation, but had no motor symptoms or signs at examination. This subject was included in the study as affected, but excluded from the neuropsychological group analyses.

**Table 2:** Clinical characteristics of SCA14 subjects

**Patient**	**Age at examination, y**	**Age at onset, y**	**SARA baseline**	**SARA 3 years follow up**	**MMS**	**MRI**
*Family 1*						
V-5	49	12	5.50	8.50	29	Cerebellar atrophy, most prominent in vermis
V-3	45	20	6.00	9.00	29	Cerebellar atrophy, most prominent in vermis
V-4	43	35	5.50	10.00	30	Cerebellar atrophy, most prominent in vermis
VI-3	20	10	10.50	12.50	29	Cerebellar atrophy
VI-1	20	10	4.00	5.00	30	**
VI-2	17	10	5.00	6.50	30	Minimal cerebellar atrophy
V-1	44	29	5.00	6.50	30	Cerebellar atrophy, most prominent in vermis
V-8	46	45	0.00	2.50	30	Cerebellar atrophy, most prominent in vermis
V-10*	54*		0	0	30	**
*Family 2*						
III-3	53	35	11.00	14.00	30	Cerebellar atrophy, some scattered unspecific white matter lesions

*This subject was without symptoms and signs, but was confirmed to have the SCA14 mutation.

**MRI not performed.

#### Controls

**Figure 1:**
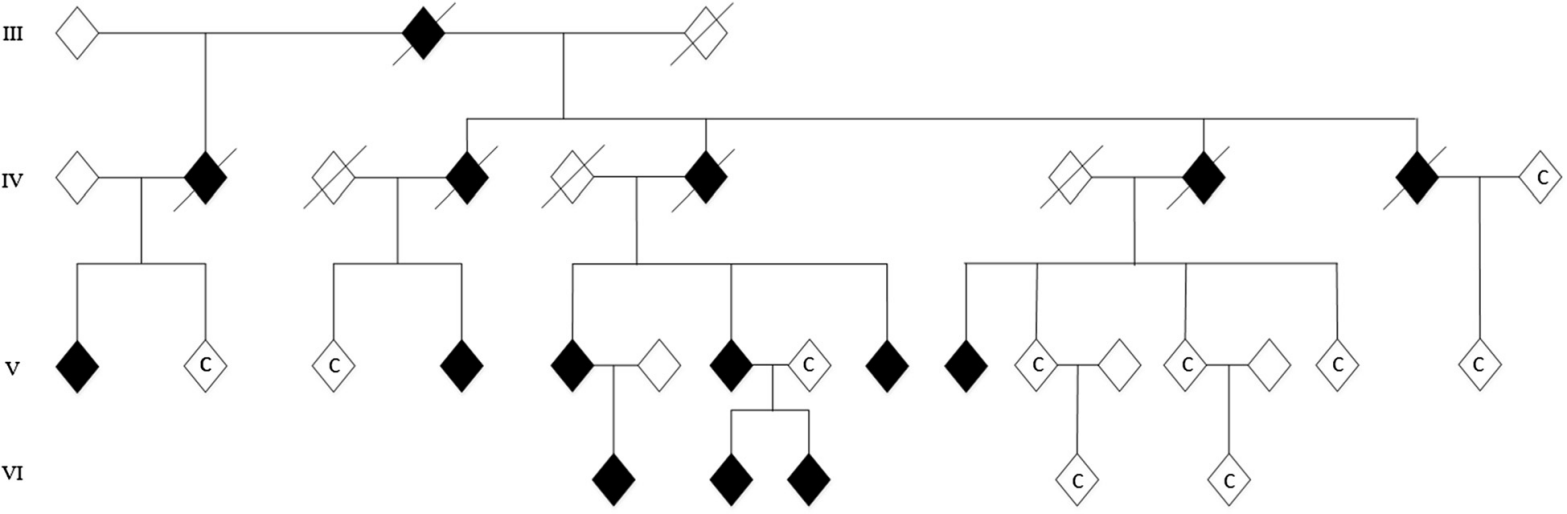
**Pedigree of SCA14 Family 1 at time of testing.** All affected living subjects were included in the study. Intrafamilial unaffected controls included in the study are marked with C. The pedigree is slightly modified due to anonymization purposes.

Ten family members, six females and four males, were included as intrafamilial controls. None of them carried the SCA14 mutation. No other neurological disease was known in these controls. They consisted of six 1st degree relatives, two 2nd degree relatives, and two spouses, and were matched for age and education (Figure 1, Table 3). The age ranged from 18 to 68 years. No intrafamilial controls were available for testing for the single affected subject in Family 2.

**Table 3:** Clinical and affective characteristics of SCA14 affected subjects and intrafamilial controls

	**Affected n = 9**	**Intrafamilial controls n = 10**
	**Median/Mean (SD)**	**Median/Mean (SD)**
SARA 1, at baseline	5.50/5.83 (3.31)	0
SARA 2, at three years follow up	8.50/8.28 (3.62)	0
Disease duration (in years)	10.00/13.00 (8.60)	0
Age at examination (in years)	44.00/37.33 (14.17)	43.5/41.9 (13.9)
Disability stage 1, at baseline	2.00/2.00 (0.50)	0
Disability stage 2, at three years follow up	2.00/2.00 (0.50)	0
Mini-Mental State (MMS)	30.00/29.67 (0.50)	0
Education (in years)	10/10.33 (2.2)	10/10.8 (2.1)
Male: Female	7:2	4:6
	**Mean (n = 9)**	Mean (n = 9)*
SCL-90		
Total	0.33 (0.41)	0.38 (0.21)
Somatization	0.71 (0.80)	0,67 (0.50)
Obsessive-compulsive	0,46 (0.69)	0,70 (0.59)
Interpersonal sensitivity	0.42 (0.43)	0.15 (0.16)
Depression	0.32 (0.48)	0.48 (0.50)
Anxiety	0.20 (0.28)	0.29 (0.25)
Hostility	0.28 (0.49)	0.11 (0.12)
Phobic anxiety	0.25 (0.43)	0.10 (0.14)
Paranoid ideation	0.22 (0.30)	0.17 (0.19)
Psychoticism	0.09 (0.14)	0.03 (0.05)
Extra-scale	0.50 (0.69)	0.83 (0.51)

*n = 9, one control was known to have ADHD and had a total SCL-90 score of 1.94, and was excluded from the analyses as an outlier.

### Clinical assessment

All affected subjects underwent a standardized neurological clinical investigation at baseline and at three years follow up. They were evaluated according to a standard clinical protocol for ataxia patients, the Scale for Assessment and Rating of Ataxias (SARA) [29]. The SARA scale has a total score from 0 (no ataxia) to 40 (most severe ataxia). A seven-stage functional scale was used to measure motor disability; 0, normal; 1, mild ataxia signs at examination, but no functional handicap; 2, mild functional disability, able to walk and run; 3, walking without help, unable to run; 4, unilateral help to walk; 5, bilateral help to walk; 6, wheelchair-bound; 7, bedridden. At three years follow up, standard brain MRI without contrast enhancement, Mini-Mental State Examination (MMS) [30] and anxiety score the Symptom Checklist-90 Revised (SCL-90-R) [31] were performed in all symptomatic subjects. A neuropsychological test panel as detailed in Table 4 was performed in all included subjects.

**Table 4:** Comparison of cognitive performance between SCA14 affected subjects and intrafamilial controls

	**Norm data**	**Affectedn = 9**	**Intrafamilial controlsn = 10**	**Affected vs norm**	**Controls vs norm**	**Affected vs controls**	Subject, V-10**	SubjectIII 3***
				***One-sample t-test***	***One-sample t-test***	***MannWhitney U test***		
	**Mean**	**Median ± interquartile range**	**Median ± interquartile range**	*p*-value*	p-value*	*p*-value*	**Scores**	**Scores**
General cognitive functioning								
Intelligence Quotient (IQ)	100	92.0 ± 10	100.5 ± 18	**0.020**	0.563	0.288	91	92
Vocabulary (t-score)	50	41.0 ± 8	44.0 ± 14	**0.001**	0.194	0.120	43	39
Matrix Reasoning (t-score)	50	53.0 ± 11	51.5 ± 18	0.479	0.602	1.000	47	52
Verbal executive function								
Controlled Word Fluency Test, Phonologic (FAS) t-score	50	45.0 ± 6	46.5 ± 9	**0.003**	0.174	0.410	43	40
Controlled Word Fluency Test, Semantic t-score	50	53.0 ± 9	53.0 ± 9	0.506	0.200	0.711	47	55
Learning and memory								
CVLT Learning percentil	50	59.0 ± 11	48.0 ± 9	0.866	0.866	**0.041**	52	65
CVLT Long-Delay Free Recall	10	14.0 ± 2	12.0 ± 2	**<0.001**	**0.005**	0.065	9	12
CVMT Learning percentil	50	10.3 ± 42.2	11.5 ± 19.3	**0.022**	**<0.001**	0.806	8.6	10.3
CVMT Memory percentil	50	27.6 ± 59.6	15.1 ± 10.8	0.278	**<0.001**	0.135	13.8	27.6
Visuoperceptive skills								
Line Orientation Test	25.4	27.0 ± 11	28.0 ± 3	0.359	0.288	0.136	19	17
Working memory								
Digit Span, sub-test, WAIS-R	10	10.0 ± 5	10.0 ± 3	0.540	0.310	0.868	8	10
Paced Auditory Serial Addition Test PASAT	46	39.0 ± 22	38.5 ± 10	**0.049**	**0.036**	0.653	****	32
Psychomotor speed								
Stroop Color Naming Test 1	10	7.0 ± 3	9.5 ± 5	**<0.001**	0.111	0.284	7	6
Stroop Color Naming Test 2	10	7.0 ± 2	7.0 ± 5	**0.003**	**0.026**	0.706	3	7
Executive functions, inhibition and set-shifting								
Stroop Color Naming Test 3	10	9.0 ± 4	9.0 ± 3	0.373	0.327	0.868	3	12
Stroop Color Naming Test 4	10	9.0 ± 4	10.5 ± 3	0.111	0.842	0.137	6	9
WCST total errors t-score	50	53.0 ± 20	38.5 ± 10	0.928	**0.005**	0.141	46	56
WCST categories	5.1	6.0 ± 1	5.5 ± 2	*****	*****	0.257	5	6
WCST conceptual level responses t-score	50	52.0 ± 20	39.0 ± 6	0.977	**0.001**	0.093	47	58
WCST perseverative responses t-score	50	55.0 ± 26	37.0 ± 12	0.661	**0.002**	0.064	49	55

*p-values < 0.05 are highlighted in bold.

**Subject V-10’s individual scores. V-10 is confirmed to carry SCA14 mutation, but was motor asymptomatic and not included in group analyses.

***Subject III-3’s individual scores. III-3 is the only subject from Family 2, and is also included in affected group analyses.

****Subject V-10 did not fulfill PASAT test.

*****not available for t-test due to not normal distribution.

All included unaffected family members were investigated clinically and screened for neurological symptoms and signs, and they were tested with the same neuropsychological tests in the same order as the affected subjects.

### Genetic testing

DNA was extracted from peripheral blood lymphocytes using standard techniques, and the subjects were identified by direct sequencing of exon 5 in the *PRKCG*-gene [19]. All healthy related intrafamilial controls were tested for the same mutation.

### Neuropsychological methods

The test panel was specifically selected to minimize motor skills’ interference and a special emphasis was put on covering executive, visuospatial and verbal domains in accordance to the CCAS. General cognitive level, psychomotor speed and memory function were also covered (Table 4). The testing lasted for two hours and was performed within standardized conditions in neurological out-patient-clinics, preferably at the subjects’ local hospital to avoid exhausting travel ahead of testing. The testing was conducted by the same neurologist (I.W.) under supervision of an experienced neuropsychologist (N.I.L). The subjects were allowed to take breaks when they wanted. The tests were scored according to standard procedures as described in the test manuals.

#### General cognitive functioning

General cognitive functioning was estimated by using the 2-subtest Norwegian version of Wechslers Abbreviated Scale of Intelligence (WASI) [32], consisting of Vocabulary and Matrix Reasoning which gives an estimate of verbal and performance IQ, respectively.

#### Memory functions

Verbal learning and memory was evaluated with California Verbal Learning Test (CVLT) [33]. A 16-word list is presented in a five-trial manner with free and delayed recall and recognition. Non-verbal learning and memory was assessed by the Continuous Visual Memory Test (CVMT) [34].

#### Visuoperceptive skills

The Line Orientation Test [35] measures the ability to judge the orientation and angles of lines in space, and requires no manual response.

#### Working memory

We used the forward and backward Digit-Span subtest from WAIS-R [36] in which the participants are presented with a series of digits and must immediately repeat them back, first in forward and then in backward direction. In addition we used the Paced Auditory Serial Addition Test (PASAT) [37,38] in which the participants hear numbers with two seconds interval and are asked to add the number they just heard with the number they heard before, requiring working memory and attention.

#### Psychomotor speed

The first two paradigms of the Stroop Color Naming Test [39] were used to give an estimate of psychomotor speed and consist of reading color names and naming color patches as rapidly as possible.

#### Executive function

Executive functions with selective attention and inhibition were tested with the Stroop Color Naming Test paradigm 3 (inhibitory) and 4 (set shifting), where the participants were asked to name the ink color of written incongruous color words as rapidly as possible, thereby assessing cognitive flexibility, selective attention and inhibitory control. To study higher executive and set-shifting functions the Wisconsin Card Sorting Test (WCST-128) [40] was used. The participants were asked to match 128 cards according to color, quantity, and design without being told the pattern on beforehand, measuring the ability to form abstract concepts and cognitive flexibility.

#### *Verbal executive functio*ns

We used The Word Fluency Test [37] which consists in rapidly saying words that starts with a specific letter F-A-S (phonologic FAS subtest) and animals and kitchen accessories (semantic subtest). The first subtest is more executively demanding and was used to evaluate verbal executive functions.

#### Depression, anxiety and somatization

Depression, anxiety and somatization were assessed by a self-report inventory, SCL-90-R [31] (Table 3).

### Norm data

All tests have internationally standardised published norms. For comparison with normal data, we used the most accurately matched available norm data for each test, adjusted to age-, education and/or sex, according to what was validated and available for the individual test.

### Data analysis

All statistical analyses were performed using SPSS for Windows version 19.0 (SPSS Inc).

Due to the small group sample, non-parametric statistical methods with Mann–Whitney U test using median as middle value were chosen when comparing the two tested groups, and the significance level was set to 0.05. After controlling for normality with Shapiro Wilk test of normality, the affected subjects’ performance using mean as middle value was compared to norm material with one-sample t-test, and the mean t-score from the norm material was used as test value. Clinical neurological progression in the three year study interval was assessed by Related Samples Wilcoxon Signed Rank Test.

Correlations between disease duration, motor symptoms, age of onset and general cognitive functioning were assessed by the non-parametric Spearman’s correlation coefficient due to the small sample size. Correction for multiple comparisons was done using the Bonferroni correction.

### Ethics

Written informed consent was obtained from all included family members (Ethical agreement n˚129/04011, Regional Ethic Committee in Norway).

## Results

All tests were conducted on all subjects except in the one asymptomatic affected subject who declined to perform the PASAT and SCL-90 tests.

### Clinical data

The duration of symptoms among the affected subjects ranged from 0 to 27 years, and there was no significant age difference between the two tested groups. (Table 2 and Table 3) SARA score in the affected subjects was significantly lower at baseline compared to the score at three-year follow-up (*p* = 0.007). Overall disability was mild with Disability Score ranging from 1 to 3, and did not vary during the study observation time. All had slowly progressive cerebellar ataxia except one who had neither symptoms nor findings at either of the two clinical examinations, and was therefore excluded from the neuropsychological group analyses. In addition, 8 subjects had pyramidal signs, consisting of extensor plantar response (6 subjects), hyperreflexia in the lower limbs (4 subjects) and increased tone in the lower limbs (5 subjects). One subject had myoclonus and one subject had head tremor.

**Figure 2:**
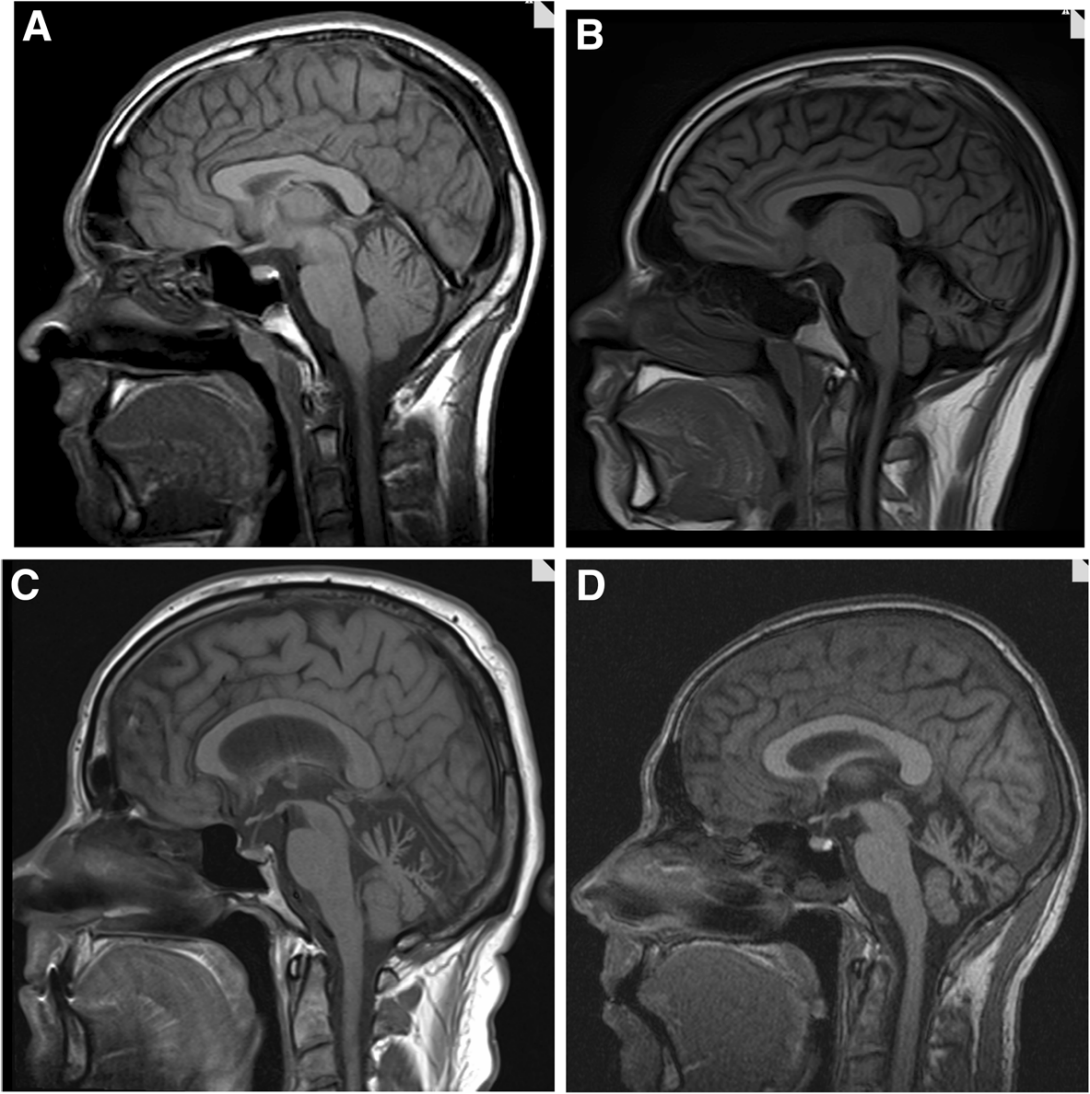
**MRI from four SCA14 subjects at time of neuropsychological testing. A**: Subject VI-2, disease duration 7 years; **B**: Subject VI-3, disease duration 10 years; **C**: Subject III-3, disease duration 18 years; **D**: Subject V-3, disease duration 25 years.

MRI was performed in eight subjects and showed isolated cerebellar atrophy in all, except in one subject where also unspecific white matter hyperintensities were described. In five of the eight subjects, atrophy was clearly most prominent in the vermis (Figure 2).

The results of the psychopathological self-report scheme SCL-90-R showed no significant differences between affected and unaffected family members, neither in total nor in any of the subcategories.

### Genetic results

The same single C to A nucleotide substitution at position 417 of exon 5 was found in the *PRKCG* gene in all affected subjects, leading to a missense mutation at the protein level, p.H139Q.

### Neuropsychological results

The affected subjects showed no significant impairment compared to intrafamilial controls in any of the tested neuropsychological domains (Table 4) but lower verbal executive function was observed. The affected performed significantly better than the intrafamilial controls in verbal memory. In the WCST the affected subjects also showed better results than the intrafamilial controls, with less perseveration and a better ability to keep their answers on a conceptual level, although it did not reach statistical significance.

Compared to population based norm data, the affected subjects showed significantly lower total IQ, verbal IQ, psychomotor speed, verbal executive function, working memory, attention and visual learning. Verbal learning was better in the affected subjects than expected from norm. However, compared to norm, lower attention, visual learning and memory, working memory and psychomotor speed were also present in the intrafamilial controls.

#### Intellectual functioning

Total and verbal IQ, but not executive IQ, were lower in affected subjects compared to the intrafamilial controls. The low estimate of verbal IQ with a mean t-score of 39.9 differed considerably from norm data and contributed to the low mean total IQ score in the affected group (*x* = 93.0). The total IQ in the affected group was significantly lower than expected from norm, even though only one affected subject scored below 1 SD from norm (*x* = 83). In verbal IQ four affected subjects scored within the clinically impaired range below 1 SD from norm (× = 40), while none of the nine affected scored above the mean expected from norm. In executive IQ seven of the nine affected scored above the mean norm expectance of 50, while only one was in the clinically impaired range. None of the affected showed a better verbal IQ than executive IQ, in contrast to four of the intrafamilial controls.

#### Memory functions

The affected subjects showed a significantly better verbal learning than the intrafamilial controls. Also compared to norm, verbal memory was significantly better among the affected subjects than expected. Six of the affected subjects showed a score 1 or more SD above what is expected in the long delay CVLT, as in contrast to only three of the intrafamilial controls.

In the CVMT both the mean t-score of visual learning (× = 14.96) and visual memory (× = 11.52) in the intrafamilial control group were lower than the affected subjects’ visual mean learning (× = 21.34) and memory (× = 33.82) t-score. In these tests the SDs were very large (visual learning SD = 29.0, visual memory SD = 35.9 in affected subjects, and visual learning SD = 16.3, visual memory SD = 6.1 in intrafamilial controls), and the difference between the groups did not reach statistical significance. However, both groups showed significant impairment compared to norm.

Regarding working memory, no significant difference was seen between the two tested groups in the PASAT-test. Both groups scored significantly below the age and IQ-matched expected mean norm score of 46, with a mean score of 36.44 for the affected, and 39.4 for the intrafamilial controls. None of the groups showed the same working memory deficit in the Digit Span Test, where there was no difference either between the tested groups or to norm.

#### Visuoperceptive skills

No difference was found between the tested groups in the Line Orientation Test. The range within the affected subjects’ group was wide with raw score performances from 17/30-30/30, and when scores were corrected for age and gender [35,41], two subjects scored within the “moderate defective” category on the 4th percentile in the norm material, but on a group level there was no difference to norm.

#### Psychomotor speed

The affected subjects performed similarly to controls in Stroop 1 + 2. Compared to norm, the affected subjects had a significantly lower mean Scaled Score in the reading (× = 6.7) and color naming (× = 7.4) subtests (Stroop 1 + 2), and the unaffected control group also scored significantly below norm in Stroop 2 (× = 7.8). In Stroop 1 only one affected subject scored similarly or above the expected Scaled Score of 10 (× = 11), while five unaffected family members did so. All affected subjects and seven of the unaffected controls scored below the Scaled Score of 10 in Stroop 2, where one affected subject also scored more than two SDs below norm (× = 3).

#### Executive functions

Verbal fluency in the affected showed no difference to unaffected family members. However, towards norm the affected scored significantly lower in the most verbal executively demanding FAS-test. All affected, and nine of the ten unaffected controls had a lower performance on the FAS-test as compared to the Semantic subtest.

Regarding non-verbal executive functions, the affected subjects performed below intrafamilial controls in Stroop 4. The single lowest performance was in the unaffected control group with a Scaled Score of 2, but seven unaffected family members scored above the expected Scaled Score of 10, while only four of the affected subjects did so. The affected tended to perform better than the unaffected controls on a trend level in two subtests of the WCST. In the total errors subtest seven of the ten affected had a t-test score above 50, in contrast to only two of the unaffected controls. There were large range and confidence intervals among the affected subjects, and both the highest (t-score 61) and lowest (t-score 33) performance of WCST total errors subtest was observed in the affected group. On a group level there was no evidence of impairment of higher executive functions among the SCA14 subjects, either towards intrafamilial controls or norm, when assessed by WCST and Stroop 3 + 4 subtests.

### Correlations between motor symptoms, duration, age of onset and cognition

The significance level was corrected for multiple testing with Bonferroni, which gave a cut-off for statistical significance of *p* = 0.00068, and no correlations were found to be statistically significant. The SARA-value at the time of neuropsychological testing (SARA2) showed a positive correlation to higher total score on the psychopathological scale SCL-90-R (r = .69, *p* = 0.041). A positive correlation was also observed between disease duration and Disability score (r = .64, *p* = 0.048). The subject with the longest duration (27 years) also had the highest total IQ performance (IQ = 107), and duration of disease showed a correlation to higher IQ on a trend level (r = .61, *p* = 0.081). There was a negative correlation between age at onset and performance in the WCST, with regard to conceptual level responses (r = −.587, *p* = 0.096) and perseverative responses (r = −.637, *p* = 0.065). The three affected subjects with the lowest disease duration (1, 7 and 8 years) were the same subjects who had the lowest WCST perseveration t-scores among the affected (t-score 36, 37 and 33).

The one somatically asymptomatic affected subject was excluded from the neuropsychological group analyses to avoid bias. Inclusion of this subject in the group assessment did not alter the overall differences between the affected subjects, either to unaffected family members or norm, and the subject’s individual results are presented in Table 4.

In the two SCA14 families tested, only one individual was available for testing in Family 2. His scores were in the similar range as the other affected subjects’ scores (Table 4).

## Discussion

These SCA14 subjects showed no statistically significant cognitive impairment to intrafamilial unaffected controls. We report verbal executive deficits in the affected subjects, but contrary to our hypothesis and to previous reports, non-verbal executive functions were not impaired; the affected subjects showed less perseveration than unaffected family members and significantly better verbal learning.

### Bias

Both affected subjects and unaffected intrafamilial controls scored below norm in several neuropsychological domains. Among the unaffected six 1st degree and two 2nd degree relatives in the control group inherited cognitive characteristics independent of ataxia may be present. The use of intrafamilial controls will reduce environmental and genetic bias. We had to include two spouses as there were no other available unaffected relatives fulfilling the age and education matching criteria. This is unfortunately the drawback of dealing with rare diseases and small families. This choice still reduces the environmental bias. However, the use of intrafamilial controls may also lead to a selection bias, as it is reasonable to assume that people choose their mates and social surroundings according to their own intellectual level and profile. In addition, the unaffected family members may be influenced by the affected, inducing a certain amount of phenocopy effect. The use of norm in addition to intrafamilial controls should reduce these confounding factors. We used international norm data, which may not fully represent the Norwegian population, especially as the Norwegian education system since 1994 has included 12 years of compulsory school education, even for artisan and craftsman qualifications. This influences the length of education for the youngest subjects in our cohort, and may give a bias towards higher scores in the norm population than in the Norwegian background population, and subseqeuently appear as an exaggeration of the impairment in the tested subjects. Thus we focus primarily on the domains where affected subjects differ from intrafamilial unaffected controls.

No single neuropsychological domain was significantly reduced compared to both norm and intrafamilial controls. This may partly be due to the choice of statistical methods. Due to the small sample size, non-parametric statistical methods were used when comparing the tested groups, as in contrast to parametric methods when comparing to norm data. We may thus have lost some statistical power, which may lead to a type II error where we falsely can have rejected a true cognitive impairment. This is however one of the main drawbacks of studies in rare disorders; our sample was small but could not be enlarged, as all identified testable and symptomatic SCA14 adult individuals in Norway were included.

In order to avoid motor bias in these ataxic patients, the test panel was selected to require minimal manual response, and the tests apparently worked well in the subjects. However, dysarthria may also affect the results. The reported psychomotor speed impairment could be due to dysarthria, but as the results only showed a subtle and not significant difference from unaffected family members in Stroop 1 + 2, we believe dysarthria is of minor importance to our results.

### Affected subjects vs. population based norm data

Compared to population-based norm data, specific aspects of cognitive function were significantly impaired in SCA14, partly consistent with the Cerebellar Cognitive Affective Syndrome (CCAS) where deficits in executive function, spatial cognition, language and affect are described. Even though the range of performance was very large, the affected showed set-shifting, planning and abstract reasoning within normal norm range, indicative of preserved non-verbal executive functions. Towards norm the affected subjects showed significantly reduced total IQ due to reduced verbal IQ, and impaired verbal executive function, attention, working memory, psychomotor speed and visual learning. Verbal memory, on the other hand, was better in the affected than expected from norm data. However, except from in IQ and verbal executive function, the unaffected family members showed the same trends as the affected towards norm. The differences can therefore hardly be due to SCA14 alone.

### Previous studies

SCA14 has been associated with cognitive impairment in previous studies (Table 1). Comparison with our results is difficult as different neuropsychological tests were used [17,25-27], and these studies have not included control groups. Earlier descriptions include primarily dysexecutive abnormalities, but also memory loss, attention deficit, frontal behaviour abnormalities, difficulty in understanding and dementia. We introduced intrafamilial controls in addition to norm, and assessed a homogeneous test panel testing broadly on different neuropsychological domains with an emphasis on domains hypothesized to be impaired in cerebellar patients. Our results do not support previous findings of severe cognitive impairment specific of SCA14. In particular, we could not confirm the earlier reported marked reduction of executive IQ and non-verbal executive functions, but we report a more pronounced verbal impairment. The differences we find compared to previous studies could partly result from genotype specificities, as the mutations reported by other groups were different from the p.H139Q mutation present in our study. Cognitive impairment in SCA14 may thus be associated with specific mutations, rather than being part of a common phenotype. As few families with SCA14 have so far been reported, genotype/phenotype correlations are, however, not yet investigated.

### Intellectual functioning

One affected subject had a clinically relevant total IQ reduction, but four had a clinically impaired verbal IQ. This indicates normal non-verbal abstract problem solving and spatial reasoning, while the ability to use language to solve problems was reduced. Only lower verbal IQ contributed to the affected group’s reduced total IQ, as compared to unaffected family members. This is in contrast to the four full-scale IQ assessments that are previously reported in SCA14 [17,25] where reduced executive IQ caused the total IQ impairment. Verbal IQ assessment is criticized for being more social and education biased than executive IQ. All the tested subjects lived in an industrial area of Norway, and only one subject in both groups had an academic education. Thus, the divergence between verbal and non-verbal abilities may be due to environmental factors in our cohort. The one affected subject with an academic background indeed had the best verbal IQ in her group (t-score = 47), but the same subject also had a comparatively higher executive IQ (t-score = 54), underscoring a lower verbal than executive IQ in these SCA14 patients, possibly due to the disease.

### Memory functions

Although visual learning and memory were similar in the tested groups, the performance range interval was remarkably large in the affected and conclusions are thus hard to draw from this small cohort. Unexpectedly, verbal learning was significantly better in the affected. Although somewhat speculative at this stage, one hypothesis could be that increased verbal memory might serve as a compensatory mechanism, mediated by impaired verbal functions in SCA14 subjects. This possibility needs to be specifically investigated in future studies.

In accordance to previous studies, we found working memory deficits towards norm in the PASAT test. However, the unaffected family members performed even lower towards norm, and we cannot interpret this as a specific SCA14 finding.

### Executive function

Contrary to our hypothesis, the only consistent evidence of dysexecutive impairment in our study was detected in verbal functions. The discrepancy in the affected subjects’ performance in the two subtests of word fluency, suggests reduced verbal executive function. This divergence cannot simply be explained by dysarthria, as impairment in both tests would be expected if dysarthria was a major component. This finding is in accordance with observations from other cerebellar disorders, where verbal fluency is one of the most consistently reported impairments [42,43].

However, non-verbal executive functions were unexpectedly weaker in the unaffected family members. Indeed the affected tended to perform below their unaffected controls in Stroop 4, but the affected subjects showed surprisingly better performances than unaffected controls with less perseveration and higher ability to find the correct sorting principles as assessed with WCST. Further studies are needed to verify these findings. It is interesting to note that also earlier studies have failed to find consistent performance differences between cerebellar patients and controls in variants of WCST [1,42,44], even though cerebellar activation has been observed during the performance of WCST in healthy subjects [45,46].

Executive dysfunction is the deficit most consistently reported previously in SCA14 patients, and may partly result from the selection of tests in these studies, with Ravens Progressive Matrix 47 [47] most widely administered [17]. This test resembles the Matrix Reasoning used in our study, but performance of Ravens Progressive Matrices is known to be adversely correlated to age and female sex [48]. The discrepancy may thus be partly due to the predominance of young male subjects in our cohort as compared to previous reports where older and more female subjects were included.

### Correlations

We hypothesized an aggravation of cognitive impairment parallel with duration of disease and increased motor disability. Unexpectedly, duration of disease showed correlation to higher IQ. Even though this correlation was not statistically significant, it is tempting to hypothesize that compensatory plasticity in the brain may develop over time in the case of early cerebellar dysfunction. An inverse correlation between age of onset and performance was also seen in WCST, where the two affected subjects with the lowest performance in the WCST had the latest age of onset. As this is a cross-sectional neuropsychological study, we have no data on the development of cognitive symptoms with duration in the individual affected subjects. The cohort is too small to draw conclusions, but we found no support for our primary hypothesis of progression of cognitive symptoms with duration. Nevertheless, plasticity would be in accordance to earlier observations of chronic cerebellar lesions leading to milder cognitive implications than acute injury [1], and cognitive impairment in acute cerebellar injury appearing to be transient.

Affective impairment is suggested by the positive correlation between motor symptoms severity and psychopathological symptoms assessed by SCL-90-R. However, the SCL-90-R also includes a scale of somatic symptoms such as fatigue and muscle pain, and by excluding the somatization scale from the analysis, the correlation weakens and one might interpret this covariance as caused by the somatic symptoms of SCA14. There remains, however, a weak correlation between motor symptoms and phobic anxiety subcategory, which could be consistent with CCAS’ prediction of dysregulated and inappropriate affect. These results will also need confirmation in larger studies, as they can be of special importance for the follow up of SCA14 patients.

All our affected subjects shared the same p.H139Q mutation in the *PRKCG* gene. Despite different genetic and environmental background, the only subject in Family 2 showed the same cognitive phenotype with the rest of the affected, with reduced verbal functions and intact non-verbal executive functions (Table 4). Interestingly, the same pattern was also observed in the motor asymptomatic subject in Family 1, and this subject performed in the lower range of the affected group (Table 4).

Although the *PRKCG* gene is expressed most abundantly in the Purkinje cells, it is expressed in the whole brain. Clinically we did observe slight pyramidal signs in some of the affected, and we cannot therefore rule out degeneration of extracerebellar neurological structures. Radiological and pathological studies in SCA14 patients are scarce, but a MRI case series showed cerebellar atrophy, and notably no cortical atrophy or white matter abnormalities [22,23]. In our study MRI was done in eight of the subjects, and isolated cerebellar atrophy was the most consistent finding (Figure 2, Table 2). This does support a primarily cerebellar explanation of the cognitive profile of this cohort.

## Conclusion

Only subtle cognitive impairment was found in SCA14 subjects compared to unaffected intrafamilial matched controls. Executive functions were not impaired to the extent that is described in the CCAS or previously reported in SCA14 patients, and impairment was restricted to verbal dysexecutive abnormalities.

Better verbal learning than intrafamilial controls may indicate possible compensatory mechanisms in affected subjects. This could point towards an interesting potential for cognitive rehabilitation for SCA14 patients. Genotype-phenotype correlations remain to be studied for cognition in SCA14. Longitudinal studies are warranted in order to confirm and strengthen the presented findings.

## Abbreviations

SCA: Spinocerebellar ataxia; PRKCG: Protein kinase C γ; ADCA: Autosomal dominant cerebellar ataxia.

## Competing interests

The authors declare that they have no competing interests.

## Authors’ contributions

IW investigated patients and controls and did the neuropsychological testing in tight collaboration and supervision from NIL, JK and CT. JK identified cases and made the clinical investigations and retesting. Study concept and design: JK, IW, CT, NIL. IW, CT and JK drafted the manuscript. All authors read and approved the manuscript.

## Pre-publication history

The pre-publication history for this paper can be accessed here:

http://www.biomedcentral.com/1471-2377/13/186/prepub
